# *In vivo* human sock‐mapping validation of a simple model that explains unipolar electrogram morphology in relation to conduction‐repolarization dynamics

**DOI:** 10.1111/jce.13606

**Published:** 2018-05-03

**Authors:** Michele Orini, Peter Taggart, Pier D. Lambiase

**Affiliations:** ^1^ Department of Mechanical Engineering University College London London United Kingdom; ^2^ Department of Cardiac Electrophysiology, The Barts Heart Center St Bartholomew's Hospital London United Kingdom; ^3^ Institute of Cardiovascular Science University College London London United Kingdom

**Keywords:** cardiac mapping, *in vivo* human data, mathematical model, repolarization, unipolar electrogram

## Abstract

**Introduction:**

The unipolar electrogram (UEG) provides local measures of cardiac activation and repolarization and is an important translational link between patient and laboratory. A simple theoretical model of the UEG was previously proposed and tested *in silico*.

**Method and results:**

The aim of this study was to use epicardial sock‐mapping data to validate the simple model's predictions of unipolar electrogram morphology in the *in vivo* human heart. The simple model conceptualizes the UEG as the difference between a local cardiac action potential and a position‐independent component representing remote activity, which is defined as the average of all action potentials. UEGs were recorded in 18 patients using a multielectrode sock containing 240 electrodes and activation (AT) and repolarization time (RT) were measured using standard definitions. For each cardiac site, a simulated local action potential was generated by adjusting a stylized action potential to fit AT and RT measured *in vivo*. The correlation coefficient (cc) measuring the morphological similarity between 13,637 recorded and simulated UEGs was cc  =  0.89 (0.72–0.95), median (Q_1_–Q_3_), for the entire UEG, cc  =  0.90 (0.76–0.95) for QRS complexes, and cc  =  0.83 (0.58–0.92) for T‐waves. QRS and T‐wave areas from recorded and simulated UEGs showed cc> 0.89 and cc> 0.84, respectively, indicating good agreement between voltage isochrones maps. Simulated UEGs accurately reproduced the interaction between AT and QRS morphology and between RT and T‐wave morphology observed *in vivo*.

**Conclusions:**

Human *in vivo* whole heart data support the validity of the simple model, which provides a framework for improving the understanding of the UEG and its clinical utility.

## 1 INTRODUCTION

The unipolar electrogram (UEG) measures cardiac extracellular potentials as the difference between the signal recorded by an exploring electrode in contact with the tissue and the potential of a remote reference often taken as the potential of the Wilson central terminal, the inferior vena cava,^1^ or other distant nonexcitable sites.^2^, ^3^

The UEG is widely used in electrophysiological research and in the catheter lab since it allows simultaneous multisite assessment of fundamental parameters such as local activation (AT) and repolarization times (RT) as well as action potential duration, tissue viability, and focal sources.^1^, ^4^, ^5^, ^6^ Indeed, current advanced mapping systems and higher density mapping electrode configurations are increasingly being utilized to provide unipolar data. However, uncertainty still remains regarding the interpretation of the UEG morphology, which can limit its application. Mathematical models have been proposed to facilitate its interpretation, but agreement with *in vivo* human data is unknown. In particular, Potse et al.^7^ have proposed a simple model that conceptualizes the UEG as the rescaled difference between a local cardiac action potential (AP) and a position‐independent component representing remote activity. This simple model was derived from a realistic multiscale 3D bidomain model, one of the most widely used theoretical models of cardiac electrophysiology,^8^ by performing mathematical simplifications based on the assumption of isotropic and homogeneous conductivity. The validity of the simple model was demonstrated *in silico* by comparing its output with that of a more complex and detailed multiscale 3D bidomain ionic model.^7^, ^9^ In the present study, we aim to provide unique human *in vivo* data to formally validate the simple model and demonstrate its validity as a useful tool to relate the morphology of the signals recorded in the catheter lab to cardiac activation‐repolarization dynamics.

We performed high‐density cardiac mapping in patients undergoing cardiac surgery using a multielectrode epicardial sock covering both ventricles from apex to the base,^2^ and we compared the morphology of UEGs recorded at each cardiac site with that of simulated UEGs generated by the simple model^7^ using as input the local AT and RT measured *in vivo* but no information regarding the UEG morphology. Results demonstrate good correlation between the morphology of simulated and recorded UEGs, suggesting that despite its simplicity the proposed model provides a sound description of the morphology of the UEGs in terms of activation‐repolarization dynamics.

## 2 METHODS

**Figure 1:**
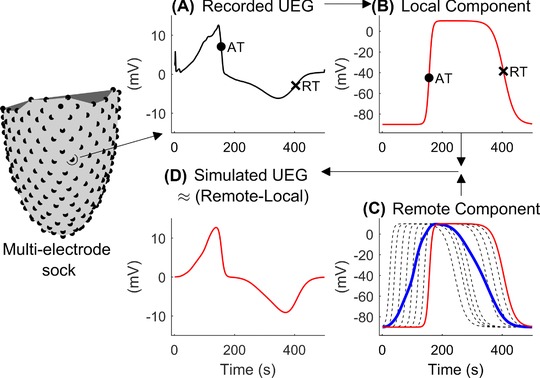
Schematic representation of the model. UEG were recorded using a multielectrode sock measuring 240 UEGs. A, Activation (AT, circle) and repolarization times (RT, cross) were measured from the unipolar electrogram (UEG). B, For each cardiac site, a local action potential (red line) was generated by adjusting a stylized action potential to fit AT and RT measured *in vivo*. C, The position‐independent component (blue bold line) representing remote activity was computed as the mean of all APs (only a representative subset of the local APs is shown dashed black lines). D, The simulated UEGs, generated as a rescaled difference between the remote and local components, shows similar morphology as the corresponding recorded one shown in panel A [Color figure can be viewed at http://wileyonlinelibrary.com]

The validation scheme implemented in this study is described in Figure 1. Local UEGs were recorded from up to 240 epicardial sites in intact human hearts during cardiac surgery and local AT and RT were measured (Figure 1A). These were used to generate stylized APs having AT and RT as measured *in vivo* (Figure 1B). A position‐independent component representing remote activity is computed as the average of all APs (Figure 1C), and finally the local UEG is obtained as a rescaled difference between the position‐independent component and the local AP (Figure 1D). Morphological features of recorded and simulated UEGs from the same site were then compared for validation. Importantly, no information related to the morphology of the UEGs recorded *in vivo* was used as model's input.

### 2.1 The simple nonionic model of the UEG

Local APs were simulated using established equations. The shape of these simulated APs is dictated by two position‐dependent parameters determining AT and RT, respectively, and two position‐independent parameters determining the steepness of the upslope and downslope during activation and repolarization, respectively. Mathematically, the local AP is the product of two logistic functions representing electrical excitation and recovery, respectively:

$$\mathrm{AP}{}_{i}\;\left(t\right)=A\frac{1}{1+e^{-\beta_{\mathrm{AT}}\left(t-\tau_{\mathrm{AT},i}\right)}}\cdot \left(1-\frac{1}{1+e^{-\beta_{\mathrm{RT}}\left(t-\tau_{\mathrm{RT},i}\right)}}\right)-V_{\mathrm{rest}}$$

In this expression, the subindex *i *= [1 … *M*] indicates a given cardiac site, the first factor represents activation and the second repolarization, *A* is the AP amplitude and $V_{rest}$ the resting potential. Parameters *β_AT_* and *β_RT_* determine the steepness of the upslope during activation and the steepness of the downslope during repolarization, respectively. Since within each heartbeat parameters *A*, *V_rest_*, *β_AT_*, and *β_RT_* are assumed to be the same for all cardiac sites (parameters are position‐independent), all APs exhibit a very similar shape. Parameters $\tau_{\mathrm{AT},i}$ and $\tau_{RT,i}$ represent local AT and RT, respectively, which in the model correspond to the time of the steepest upslope during activation,${\left(dAP_{i}\left(t\right)/dt\right)}_{\max}$, and the time of the steepest downslope during repolarization, ${\left(dAP_{i}\left(t\right)/dt\right)}_{\min}$, respectively. Following what was proposed by Potse et al.,^7^ the remote component is defined as the average AP computed among all cardiac sites:

$$\bar{\mathrm{AP}(t)}=\frac{1}{M}\;\sum_{i\;=\;1}^{M}\mathrm{AP}{}_{i}\left(t\right)$$

The local UEG measured at a given site *i* is obtained by inverting and rescaling the difference between the local AP and the position‐independent remote component (see Figure 1):

$$\mathrm{UEG}{}_{i}\;\left(t\right)=\;-\alpha \left(\mathrm{AP}{}_{i}\left(t\right)-\bar{\mathrm{AP}(t)}\right)$$

The scaling factorα is equal to $\alpha \;=g_{i}/\left(g_{i}+g_{e}\right)\;$ and represents the balance between intracellular ($g_{i}$) and extracellular ($g_{e}$) conductivities, which are assumed to be constant in space and time.^7^ Mathematically, it is possible to demonstrate that within this theoretical framework, standard measures of AT and RT derived from UEGs, usually timed to ${\left(dUEG(t)/dt\right)}_{\min}$ and ${\left(dUEG(t)/dt\right)}_{\max},$ respectively, are equal to local AT, $\tau_{\mathrm{AT},i}$, and RT, $\tau_{RT,i}$, as long as the remote component $\bar{AP(t)}$ changes much more slowly than the local AP, $AP_{i}\left(t\right)$. The amplitude of the QRS complexes and T‐waves depends on parameters *β_AT_* and *β_RT_*, as well as on spatial dispersion of both depolarization and repolarization.

**Figure 2:**
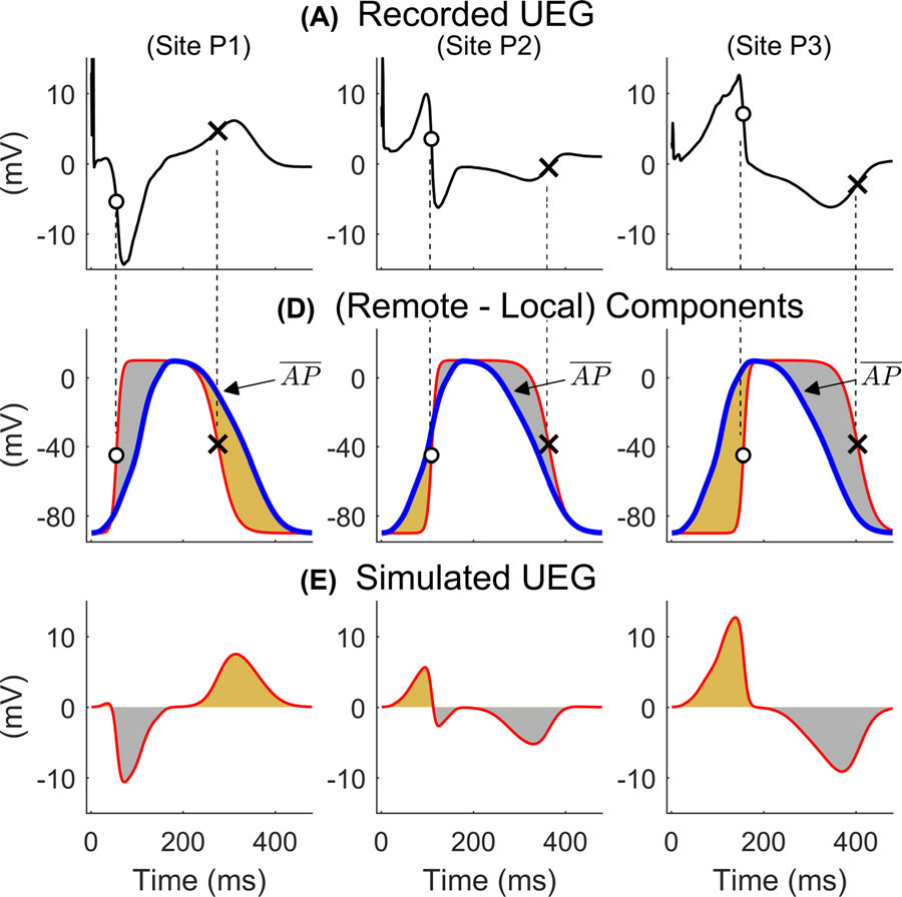
Top: UEGs recorded in one patient at sites P_1_–P_3_ showing different morphologies. Middle: Local (bold line) and remote position‐independent (blue bold line) components from the simple model. Bottom: Simulated UEGs generated as a rescaled difference between the remote and local components are similar to the recorded one (top panels). From left to right, a UEG shows QS‐, RS‐, or R‐waves when the local component precedes, intersects, or follows the remote one, respectively. Similarly, a UEG shows a positive, biphasic, or negative T‐wave when the local component precedes, intersects, or follows the remote one, respectively. The occurrence of QRS complexes and T‐waves with different morphologies depends on activation and repolarization sequences, respectively, and any combination is possible. Figures adapted from (7) [Color figure can be viewed at http://wileyonlinelibrary.com]

Importantly, as shown in Figure 2, the model predicts that sites that activate before (or after) the remote component exhibit QS complexes (or prominent R waves), and sites that repolarize before (or after) the remote component exhibit a positive (or negative) T‐wave.

### 2.2 Clinical study and data analysis

Whole‐heart epicardial contact mapping was performed during open‐heart cardiac surgery using a multielectrode sock,^2^, ^10^ enabling the acquisition of 240 UEGs. The multielectrode sock was made of a flexible material allowing to fit over hearts of different sizes and cover both ventricles from apex to base. Eighteen patients undergoing either coronary artery bypass grafting (n = 14) or aortic valve replacement (n = 1) or both (n = 3) were studied. The study was approved by the local Ethics Committee and all patients gave written informed consent. S1 drive trains of 30–50 beats were delivered from one of the epicardial electrodes at cycle lengths decreasing from 600 to 350 ms. UEGs were recorded at a sampling rate of 1 KHz within 0.05–500 Hz, and referenced to the rib retractor. Hemodynamic stability during the pacing protocol was closely monitored and pacing discontinued if appropriate. Data were exported and analyzed offline with bespoke algorithms as in previous studies.^10^, ^11^, ^12^ Signal averaging of beats with same cycle length was conducted to reduce background noise. UEGs showing beat‐to‐beat morphological variability, i.e., with mean correlation coefficient between the median UEG and UEGs from any single beat lower than 0.98, were not considered. Signal‐to‐noise ratio (SNR) was assessed using a spectral‐based measure where spectral bands for signal and noise were defined within 1–40 Hz and 40–100 Hz, respectively. Signal‐averaged UEGs with SNR < 10 dB were not considered. AT and RT were measured on the signal‐averaged UEGs using standard definitions, i.e., AT and RT was measured as the intervals between the pacing stimulus and the time of the minimum of the first derivative during the QRS complex and the maximum of the first derivative of the T‐wave independently of its polarity,^13^ respectively. Activation recovery interval (ARI), a standard surrogate for the local AP duration,^3^ was measured as ARI = RT – AT.

For each beat, the local AT and RT measured *in vivo* were used to generate corresponding local APs showing the same AT and RT, i.e., by modifying $\tau_{\mathrm{AT},i}$ and $\tau_{RT,i}$ (Figure 1B). The pair of position‐independent parameters {*β_AT_*, *β_RT_*} was chosen as that providing the highest median correlation coefficient (best morphological matching) between real and simulated UEGs among all combinations of *β_AT _*= {0.2, 0.4, 0.6} ms^−1^ and *β_RT_*_ _= {0.025, 0.035, 0.045, 0.055} ms^−1^, while *α* = 0.25 in all configurations.

### 2.3 Statistical analysis

The Pearson's correlation coefficient was used to assess the similarity between recorded and simulated AT and RT sequences, as well as between morphological features of recorded and simulated UEG from the same cardiac site. The morphological similarity was assessed considering the entire UEGs, as well as the QRS complex and T‐wave separately. Standard box‐plots were used to describe data distribution, where central line is the median, the edges of the box are the first (Q_1_) and third (Q_3_) quartiles, and the whiskers extend to the most extreme data points not considered outliers. Values lower than Q_1_ – 1.5*(Q_3_ – Q_1_) and higher than Q_3_ + 1.5*(Q_3_ – Q_1_) are considered outliers. Nonnormally distributed data are described by the median, median absolute deviation, i.e., median(X‐median(X)), and interquartile range. Statistical analysis was performed both pooling the data together and on a patient‐by‐patient basis.

## 3 RESULTS

In total, after applying the aforementioned automatic inclusion criteria based on morphological stability and signal quality, 13,637 UEGs from 18 patients were utilized (Table 1).

**Table 1:** Results showing similarity between data recorded *in vivo* in‐human (R) and model data (M) grouped by cycle length

	350	400	450	500	550	600
	Cardiac intervals					
ΔAT (R‐M)	0±0	0±0	0±0	0±0	0±0	0±0
cc‐AT (R‐M)	1±6e‐06	1±9e‐06	1±7e‐06	1±9e‐06	1±1e‐05	1±1e‐05
N	158±15	160±19	166±18	167±19	171±24	165±21
ΔRT (R‐M)	−4±3	−1±3	−1±1	−1±1	0±1	0±1
cc‐RT (R‐M)	0.99±0.004	0.99±0.003	1±0.002	1±0.001	1±0.001	1±0.001
N	149±16	155±23	166±18	167±19	171±24	165±21
ΔARI (R‐M)	−4±3	0±3	0±1	0±1	0±0.5	0±2e‐13
cc‐ARI (R‐M)	0.81±0.1	0.9±0.04	0.95±0.03	0.95±0.03	0.93±0.06	0.94±0.05
N	149±16	155±23	166±18	167±19	171±24	165±21
	Morphological correlation					
N	153±17	161±19	167±15	171±23	179±25	177±13
cc‐UEG (R‐M)	0.89±0.02	0.9±0.05	0.9±0.05	0.85±0.05	0.81±0.06	0.81±0.06
cc‐TW (R‐M)	0.95±0.02	0.85±0.04	0.81±0.05	0.81±0.04	0.81±0.04	0.8±0.07
cc QRS (R‐M)	0.89±0.03	0.89±0.05	0.89±0.05	0.89±0.05	0.88±0.06	0.89±0.04
	Morphological features					
cc QRSa (R‐M)	0.89±0.05	0.90±0.02	0.89±0.02	0.91±0.03	0.92±0.03	0.91±0.04
cc TWa (R‐M)	0.84±0.06	0.88±0.05	0.87±0.03	0.88±0.04	0.87±0.08	0.88±0.07
cc AT‐QRSa (R)	0.89±0.04	0.91±0.02	0.9±0.01	0.91±0.02	0.92±0.02	0.91±0.03
cc AT‐QRSa (M)	0.99±0.003	0.99±0.003	0.99±0.002	0.99±0.002	1±0.002	1±0.002
cc RT‐TWa (R)	−0.87±0.05	−0.89±0.04	−0.86±0.03	−0.88±0.05	−0.89±0.07	−0.89±0.06
cc RT‐TWa (M)	−0.99±0.003	−1±0.002	−1±0.002	−1±0.001	−1±0.002	−1±0.001

*Note*: ΔAT, ΔRT and ΔARI: median differences between recorded and simulated cardiac intervals. cc‐AT, cc‐RT and cc‐ARI: correlation coefficients between ATs, RTs and ARI from recorded and simulated data. ccAT‐QRSa and ccRT‐TWa: correlation coefficients between AT and the QRS area, and between RT and T‐wave area, within recorded and model data. cc‐QRSa and cc‐TWa: correlation coefficients comparing the QRS area and T‐wave area in the recorded and simulated data, respectively. All results are given as median ± median absolute deviation.

### 3.1 Activation and repolarization sequence

As expected owing to the modeling design, AT and RT measured from the simulated UEGs were very similar to those measured from UEGs recorded *in vivo*, with correlation coefficients higher than 0.97 and minimal absolute differences (Table 1).

### 3.2 Morphological features

**Figure 3:**
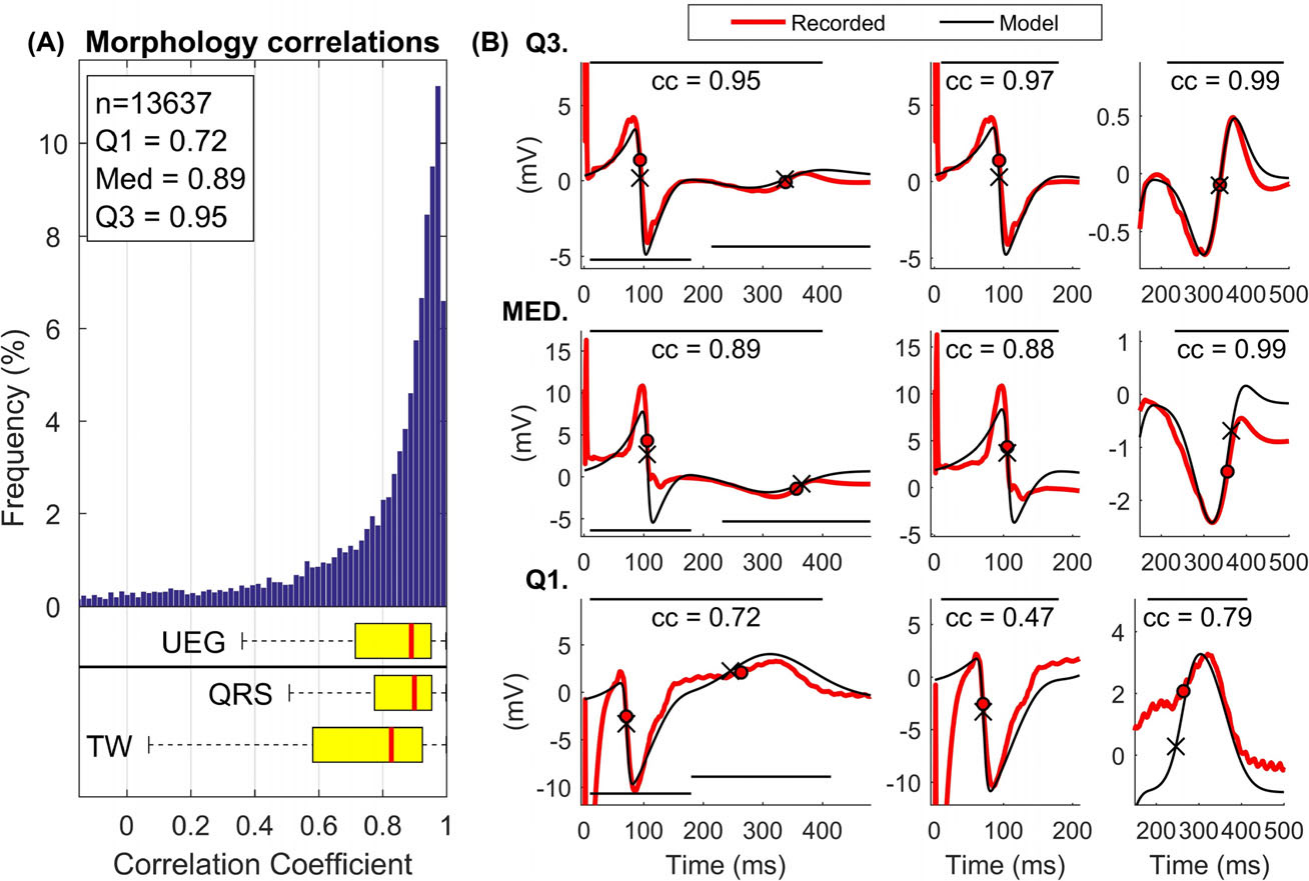
A, Distribution of the correlation coefficients indicating the morphological similarity between recorded and simulated UEGs. Correlations were considered within the entire signal (indicated as UEG in the figure) as well as within the QRS and the T‐wave, separately. B, Representative examples of recorded (red) and simulated (black) UEGs corresponding to correlation coefficients equal to the first (Q1), second (MED), and third (Q3) quartile. The correlation coefficients are calculated within the entire signal (left), within the QRS (middle), and the T‐wave (right), and the panels are adjusted to highlight the correlation within these intervals [Color figure can be viewed at http://wileyonlinelibrary.com]

The morphology of the simulated UEGs was very similar to the morphology of the corresponding UEGs recorded *in vivo*. As shown in Figure 3A, pooling all UEGs together, the median correlation coefficient between simulated and recorded UEGs was equal to cc = 0.89 (Q_1_/Q_3 _= 0.72/0.95, n = 13,637). This demonstrates that the morphological similarity between simulated and recorded UEGs was good (cc> 0.72) in at least 75% and excellent (cc > 0.95) in at least 25% of all recordings, while only less than 25% of simulated UEG showed moderate correlation (cc < 0.72) but still reproduced the most relevant morphological features in terms of QRS and T‐wave polarity. The correlation coefficient was higher for the QRS complex (median 0.90, Q_1_/Q_3 _= 0.78/0.95) than for the T‐wave (median 0.83, Q_1_/Q_3 _= 0.58/0.92) (P < 0.001). Similar results were observed when patient‐by‐patient analysis was conducted (Table 1). In this case, the median correlation coefficients between simulated and recorded UEGs was slightly higher for the QRS complex than for the T‐wave for all cycle lengths except 400 ms and 600 ms for which only a trend was observed (0.10 < P < 0.05, Table 1). The first column of Figure 3B shows representative recorded and simulated UEGs exhibiting correlation coefficients equal to the first (bottom), second (middle), and third (top) quartile of the entire distribution. Morphological comparison of QRS complexes and T‐waves from the same recordings is highlighted in the second and third columns, respectively.

**Figure 4:**
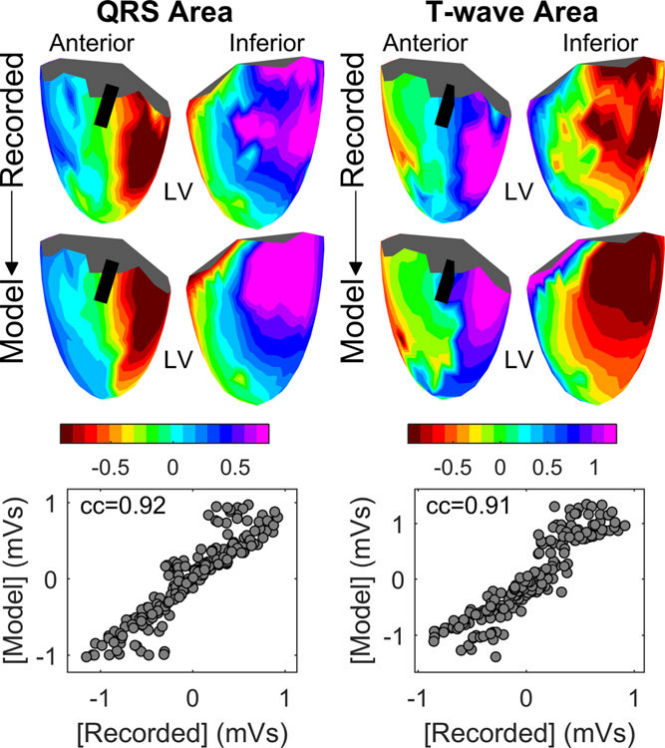
Representative example of morphological similarity between recorded and simulated unipolar electrograms (UEG). Color maps show the area under the QRS complexes (left) and T‐waves (right) of UEGs recorded in one patient (top, anterior view on the left, inferior view on the right) and in the corresponding simulated UEGs (below). The black line on the ventricular mesh indicates the approximate location of the LAD. On the bottom, the scatterplots indicate high correlation between the recorded and simulated QRS area (left) and T‐waves (right) (correlation coefficient cc ≥ 0.91) [Color figure can be viewed at http://wileyonlinelibrary.com]

Furthermore, the correlation between the area under the QRS complex of recorded and simulated UEGs was cc> 0.89 for all CLs, while the correlation between the area under the T‐wave of recorded and simulated UEGs was cc> 0.84 for all CLs (Table 1), indicating an almost perfect match between voltage isochrones maps from recorded and simulated data. A representative example of maps showing QRS and T‐waves areas from both recorded and simulated data is shown in Figure 4.

**Figure 5:**
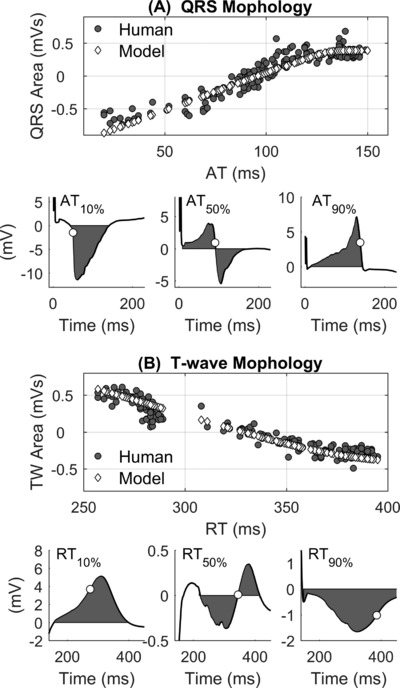
Spatial interaction between activation/repolarization dynamics and the morphology of the unipolar electrograms. A, Strong positive correlation between the area under the QRS complex of the local unipolar electrogram (UEG) and local activation (AT) measured at the same site. Gray circles and white diamonds indicate results from recorded and simulated UEGs, respectively. Bottom: examples of QRS complexes from recorded UEGs corresponding to AT equal to the 10th, 50th, and 90th percentile (white circles). B, Strong negative correlation between the area under the T‐wave of the local unipolar electrogram (UEG) and local repolarization time (RT) measured at the same site. Gray circles and white diamonds indicate results from recorded and simulated UEGs, respectively. Bottom: examples of T waves from recorded UEGs corresponding to RT equal to the 10th, 50th, and 90th percentile (white circles)

*In vivo* data analysis demonstrates that the interactions between AT distribution and QRS morphology as well as between RT distribution and T‐wave morphology described in Figure 2 and predicted by the model exist in the intact human heart. Figure 5A shows that in both recorded and simulated UEGs, cardiac sites that activate early exhibit a QS complex, those that activate around the mean ATs exhibit an RS complex with positive and negative deflections of similar amplitude while those that activate late exhibit a prominent R wave. Similarly, the T‐wave is positive for sites that repolarize early, bipolar for sites whose RT is approximately equal to the mean RT and negative for sites that repolarize late (see Figure 5B). Therefore, a positive correlation exists between AT and the area under the QRS complex, while a negative correlation exists between RT and the area under the T‐wave (see Figure 5A and B). These correlations were observed in all patients for all cycle lengths (Table 1), with a median correlation coefficients between AT and QRS area and between RT and T‐wave area approximately equal to 1 and –1, respectively, in simulated UEGs, and approximately equal to 0.9 and –0.9, respectively, in human UEGs.

## 4 DISCUSSION

The simple model described in this article and first proposed by Potse et al.^7^ explains the morphology of the UEG as the difference between the local AP and a position‐independent remote component, which is equal to the mean AP. Although the model has been previously tested *in silico*,^7^ it still lacked formal experimental validation. This article provides its first *in vivo* in‐human validation. The comparison with more than 13,000 UEGs recorded during whole‐heart epicardial sock mapping in 18 patients demonstrates that this analytical nonionic framework accurately reproduces: (1) The morphology of the entire UEG as well as the morphology of the QRS complex and T‐wave, with median correlation coefficients equal to 0.89, 0.90, and 0.83, respectively (Figure 3); (2) Morphological features such as QRS and T‐wave areas, with median correlation coefficients higher than 0.89 and 0.84 (Table 1), respectively, indicating an almost perfect match between voltage isochrones maps from recorded and simulated data (Figure 4); (3) *In vivo* interaction between local AT and QRS morphology as well as between local RT and T‐wave polarity (Figure 5). A close match observed between simulated and recorded AT, RT, and APD sequences was expected owing to the model design. Overall, these results show that the simple model accurately explains the fundamental link between the morphology of the UEGs and activation/repolarization dynamics. It is worth mentioning that the striking similarity between recorded and simulated UEGs is by no means the consequence of a circular reasoning, as no information related to the UEG morphology was used in the model. Furthermore, the model was kept as simple as possible to avoid overfitting: the shape of the simulated APs was forced to be very similar, only two logistic functions were used to simulate the APs instead of three as in previous studies,^14^ parameters *β_AT_* and *β_RT_* were assumed position‐independent and allowed to take only few prespecified values.

Potse et al.^7^ derived the simple model by simplifying a realistic multi‐scale 3D bidomain model, one of the most widely used theoretical model of cardiac electrophysiology,^14^ based on the assumption that the conductivity tensor fields of both intracellular and extracellular domains were isotropic and homogeneous. In that study,^7^ the local component of the UEG corresponded to the local AP generated using a state of the art computational model including ion‐currents dynamics.^9^, ^15^ The T‐wave of simulated UEGs closely correlated to those generated implementing the more complex and realistic model, and the results were used to support the Wyatt method for RT assessment. In this work, the implementation of the simple model by Potse et al.^7^ was further simplified by using as local components mathematical functions providing a stylized AP whose upstroke and down‐slope are adjusted to match local AT and RT. A similar approach has been adopted in previous theoretical studies.^14^, ^16^, ^17^ Of note, in this study we found a high morphological correlation between recorded and simulated UEG even if the model was informed with only epicardial data. This does not imply that endocardial and septal activity do not contribute to the morphology of epicardial UEG, but it is most likely due to the fact that sock‐mapping of both the left and right ventricles provided an accurate measure of the average AT and RT and therefore a reliable evaluation of the position‐independent component representing remote activity, which is fundamental to determine the shape of the local UEG. Furthermore, since the simple model assumes homogenous and isotropic conductance, it is best suited to represent UEGs in normal cardiac tissue and its validity in presence of scar should be investigated in future studies.

The possibility of performing multisite RT measurements is a unique feature of the UEG that offers the opportunity of investigating a number of physiological mechanisms linked to ventricular arrhythmia such as spatial inhomogeneity of repolarization,^18^ repolarization alternans and variability,^19^, ^20^ cardiac restitution,^10^, ^21^ and activation‐repolarization coupling.^22^, ^23^ The investigation of these mechanisms, along with the understanding of the UEG morphology, is critical for prediction as well as ablation of ventricular tachycardias. Indeed, the increased resolution of high‐density mapping technologies and new mapping electrode configurations now enable finer mapping of the substrate utilizing unipolar data, so correct interpretation of this information is even more pertinent in the modern electrophysiology arena.^24^

Extensive work^3^, ^7^, ^25^, ^26^, ^27^, ^28^ has consistently demonstrated that the time of the steepest upslope during the T‐wave is a reliable marker of local RT, as first suggested by Wyatt et al.^13^ The simple model predicts that upright and inverted T‐waves are associated with early and late repolarization, respectively, and its validation in the *in vivo* human heart provides further support to the Wyatt method.

The power of this model lies in its simplicity, as it explains the UEG morphology as the difference between only two components, one position‐dependent and similar to the local AP and another position‐independent and similar to the average of all APs, representing remote activity. This model could be used to study mechanisms and predict outcome as it provides a simple conceptual framework for linking properties of the UEG to the underlying activation and repolarization spatiotemporal dynamics, generate and test hypotheses, assess new methodologies, and ultimately improve the interpretation and clinical utility of the UEG. Similar analytical approaches have been previously used for similar purposes.^7^, ^29^, ^30^, ^31^, ^32^, ^33^

## 5 CONCLUSIONS

The similarity between more than 13,000 UEGs recorded with a multielectrode epicardial sock in 18 patients and UEGs generated by the simple theoretical model implemented in this study and first proposed by Potse et al.^7^ demonstrates that (1) the UEG can be conceptualized as the difference between a local cardiac AP and a position‐independent component representing remote activity and that (2) its morphology is mainly determined by activation‐repolarization dynamics. This has important implications in the interpretation of high‐density mapping data to advance understanding of arrhythmia mechanisms and enable optimal ablation strategies.
